# Editorial: Sexual Dimorphism in Glucose and Lipid Metabolism

**DOI:** 10.3389/fendo.2016.00166

**Published:** 2016-12-23

**Authors:** Janne Lebeck

**Affiliations:** ^1^The Danish Diabetes Academy Supported by the Novo Nordisk Foundation, Odense, Denmark; ^2^Department of Biomedicine, Aarhus University, Aarhus, Denmark; ^3^Department of Clinical Biochemistry, Aarhus University Hospital, Aarhus, Denmark

**Keywords:** sex, lipid metabolism, glucose metabolism, skeletal muscle, adipose tissue, liver, sex hormones

**Editorial on the Research Topic**

**Sexual Dimorphism in Glucose and Lipid Metabolism**

Obesity and especially central obesity is associated with increased risk for development of type 2 diabetes and cardiovascular disease. Importantly, men have a greater amount of visceral adipose tissue than premenopausal women (Lundsgaard and Kiens; Varlamov et al.) and are more prone to develop pathological alterations in glucose and lipid metabolism (Lundsgaard and Kiens). Thus, understanding how sex influences energy metabolism might hold key answers to how obesity related diseases can be prevented. Therefore, the aim of this research topic is to give an overview of the current knowledge on sexual dimorphisms in glucose and lipid metabolism. The articles are mainly focused on knowledge obtained in humans using different tracer studies. Overall, the data point toward a complex role of sex hormones where the sexual dimorphisms depend on the metabolic status of the individual. This includes that women in the prandial and postprandial state utilize more carbohydrate than men to meet energy needs (Lundsgaard and Kiens), whereas during prolonged fasting or exercise women rely more on fat oxidation than men (Lundsgaard and Kiens; Varlamov et al.; Hedrington and Davis; Henderson; Santosa and Jensen) (summarized in Table 1).

**Table 1:** **Summary of the reported differences in metabolism in fed and fasted men and women**.

	Fed		Fasted	
	Women	Men	Women	Men
Adipose tissue	Meal FFA storage: femoral > abdominal SAT (Santosa and Jensen)	Meal FFA storage: femoral = abdominal SAT (Santosa and Jensen)	Direct FFA storage: femoral > abdominal SAT (Varlamov et al.; Santosa and Jensen)	Direct FFA storage: femoral < abdominal SAT (Varlamov et al.; Santosa and Jensen)
	↑↑ Glucose uptake (Lundsgaard and Kiens)	↑ Glucose uptake (Lundsgaard and Kiens)	↑↑ Lipolysis (prolonged fasting) (Hedrington and Davis)	↑ Lipolysis (prolonged fasting) (Hedrington and Davis)
	↑↑ TG synthesis in SAT (Varlamov et al.)	↑ TG synthesis in SAT (Varlamov et al.)	↑ Post-exercise lipolysis (Henderson)	↑↑ Post-exercise lipolysis (Henderson)
Skeletal muscle	Morphology: ↑ type 1 fibers and greater capillary density/muscle area (Lundsgaard and Kiens)	Morphology: ↑ type IIA and IIX fibers and lower capillary density/muscle area (Lundsgaard and Kiens)	Morphology: ↑ type 1 fibers and greater capillary density/muscle area (Lundsgaard and Kiens)	Morphology: ↑ type IIA and IIX fibers and lower capillary density/muscle area (Lundsgaard and Kiens)
	↑↑ Glucose uptake (Lundsgaard and Kiens)	↑ Glucose uptake (Lundsgaard and Kiens)	↑↑↑ VLDL-TAG clearance (Santosa and Jensen)	↑ VLDL-TAG clearance (Santosa and Jensen)
	↑↑ IM-TAG stored in small lipid droplets with ↑↑ perilipin (Lundsgaard and Kiens)	↑ IM-TAG stored in larger lipid droplets with perilipin (Lundsgaard and Kiens)	↑↑ CD36 protein abundance (Lundsgaard and Kiens)	↑ CD36 protein abundance (Lundsgaard and Kiens)
	↑↑ Capacity for β-oxidation (Lundsgaard and Kiens; Hedrington and Davis)	↑ Capacity for β-oxidation (Lundsgaard and Kiens)	↑↑ TAG storage (Lundsgaard and Kiens)	↑ TAG storage (Lundsgaard and Kiens)
			↑↑ IM-TAG usage (Lundsgaard and Kiens)	↑ IM-TAG usage (Lundsgaard and Kiens)
			↑↑ β – oxidation during exercise (Henderson)	↑↑ Carbohydrate oxidation during exercise (Henderson)
			↑ Rate of post-exercise β – oxidation (if fasted) (Henderson)	↑↑ Rate of post-exercise β – oxidation (if fasted) (Henderson)
			↑ Capacity for glycolysis (Lundsgaard and Kiens; Hedrington and Davis)	↑↑ Capacity for glycolysis (Lundsgaard and Kiens)
			↑ Sympathetic nerve activity (Hedrington and Davis)	↑↑ Sympathetic nerve activity (Hedrington and Davis)
			↑ Glycogen depletion (Varlamov et al.)	↑↑ Glycogen depletion (Varlamov et al.)
Liver			↑↑ VLDL-TAG secretion (Henderson; Santosa and Jensen)	↑ VLDL-TAG secretion (Henderson; Santosa and Jensen)
			↑ TAG storage (Lundsgaard and Kiens)	↑↑ TAG storage (Lundsgaard and Kiens)
			↑ HGP (Varlamov et al.; Hedrington and Davis)	↑↑ HGP (Varlamov et al.; Hedrington and Davis)
			↑ Glycerol permeability of hepatocyte plasma membrane in obese with NAFLD (Rodriguez et al.)	↑↑ Glycerol permeability of hepatocyte plasma membrane in obese with NAFLD (Rodriguez et al.)
Plasma	↑ VLDL-TAG levels (Lundsgaard and Kiens; Santosa and Jensen)	↑↑ VLDL-TAG levels (Lundsgaard and Kiens; Santosa and Jensen)	↑ VLDL-TAG levels (Lundsgaard and Kiens; Santosa and Jensen)	↑↑ VLDL-TAG levels (Lundsgaard and Kiens; Santosa and Jensen)
	↑↑↑ Leptin (Lundsgaard and Kiens; Hedrington and Davis)	↑ Leptin (Lundsgaard and Kiens; Hedrington and Davis)	↑↑↑ Adiponectin (Lundsgaard and Kiens)	↑↑↑ Adiponectin (Lundsgaard and Kiens)
	↑↑↑ Adiponectin (Lundsgaard and Kiens)	↑ Adiponectin (Lundsgaard and Kiens)	↑↑ FFA (Lundsgaard and Kiens; Hedrington and Davis)	↑ FFA (Lundsgaard and Kiens; Hedrington and Davis)
			↑↑ Glycerol (Hedrington and Davis)	↑ Glycerol (Hedrington and Davis)
			↑↑ Total LPL activity (Lundsgaard and Kiens; Varlamov et al.)	↑ Total LPL activity (Lundsgaard and Kiens; Varlamov et al.)
			↑ Epinephrine, norepinephrine, glucagon, GH, PP (Hedrington and Davis)	↑↑ Epinephrine, norepinephrine, glucagon, GH, PP (Hedrington and Davis)
			↓↓ Glucose (prolonged fasting) (Hedrington and Davis)	↓ Glucose (prolonged fasting) (Hedrington and Davis)
			↑ Postprandial lipemia (lean) (Henderson)	↑↑ Postprandial lipemia (Henderson)
			↑↑ Rapid normalization of plasma glucose after exercise (Henderson)	↑ Rapid normalization of plasma glucose after exercise (Henderson)

*Direct FFA storage: a portion of FFA form adipose tissue lipolysis is stored in distant adipose tissue*.

*SAT, subcutaneous adipose tissue. IM-TAG, intramyocellular triacylglycerol; HGP, hepatic glucose production; LPL, lipoprotein lipase; GH, growth hormone; PP, pancreatic polypeptide; VLDL, very low density lipoproteins*.

## Glucose

Apart from the sexual dimorphism in when carbohydrates are preferred to meet energy needs, there are also sex differences in blood glucose levels (Hedrington and Davis). As reviewed by Hedrington and Davis, women endure lower blood glucose levels than men during prolonged fasting. This seems a consequence of a blunted central nervous system efferent response that in turn results in lower plasma levels of counter regulatory hormones and lower hepatic glucose output (Hedrington and Davis).

## Lipid

As reviewed by Santosa and Jensen, there are also substantial differences in lipid metabolism between men and women. Regional differences in FFA storage (Varlamov et al.; Santosa and Jensen) seem to contribute to the android (upper body)/gynoid (lower body) fat distribution (Table 1), whereas the role of depot differences in lipolytic rate is less clear (Varlamov et al.; Santosa and Jensen). However, there are sex differences in adipose tissue lipolysis during fasting and exercise, where women have a higher whole body lipolytic rate and higher plasma FFA levels than men (Lundsgaard and Kiens; Hedrington and Davis; Santosa and Jensen). In the postabsorptive state, most plasma triacylglycerol (TAG) is found in very low density lipoproteins (VLDL) secreted by the liver. Women have lower plasma levels of VLDL-TAG than men, which is due to a higher clearance rate of TAG by skeletal muscle (Lundsgaard and Kiens; Santosa and Jensen).

## Insulin Sensitivity

Insulin sensitivity is often measured as whole body insulin sensitivity and both no difference between men and women, and women having higher whole body insulin sensitivity than men have been reported (Varlamov et al.; Hedrington and Davis). Importantly, as reviewed by Lundsgaard and Kiens, the evidence for higher insulin sensitivity in female skeletal muscle stands stronger than the potential sexual dimorphism in whole body insulin sensitivity. Most studies investigating sex differences in insulin sensitivity have been conducted in healthy, lean individuals, thus leaving its significance in obese a largely unanswered question (Ter Horst et al.). In the study by Ter Horst et al., severely obese men had lower hepatic insulin sensitivity than BMI and age matched women, suggesting that targeting the liver when treating type-2-diabetes is particularly important in men.

## The Role of Sex Hormones

How sex influences energy metabolism is highly complex and as pointed out in the review by Varlamov et al., even the gene-dosage effects of X and Y chromosomes should be taken into account. In addition to the effect on peripheral tissues, estrogen also directly influences the central nervous system and promotes anorexia through actions in hypothalamus (Varlamov et al.). Overall both estradiol and testosterone deficiency affects energy metabolism in a manner suggesting that both hormones have a protective role against obesity and insulin resistance (Varlamov et al.), with estradiol having the most pronounced effects (Santosa and Jensen). However, excess of both hormones, such as seen in women using oral contraceptives (excess exogenous estrogen) or women with PCOS (excess androgen), promotes insulin resistance (Lundsgaard and Kiens), and the beneficial effect of sex hormone repletion is generally unresolved (Santosa and Jensen).

In all, the articles underline the significance of sex differences in energy metabolism and show the importance of studying obesity related diseases in a sex-specific manner as conclusions made in one-sex studies only apply to that particular sex. Furthermore, much knowledge can be gained by understanding why premenopausal women despite a higher fat mass, remains less susceptible to obesity related diseases.

## Author Contributions

The author confirms being the sole contributor of this work and approved it for publication.

## Conflict of Interest Statement

The author declares that the research was conducted in the absence of any commercial or financial relationships that could be construed as a potential conflict of interest.

